# Recurrent hydrothorax in a child on peritoneal dialysis: A case report and review of the literature

**DOI:** 10.1002/ccr3.1936

**Published:** 2018-11-25

**Authors:** Khalid A. Alhasan

**Affiliations:** ^1^ Pediatric Department King Saud University College of Medicine Riyadh Saudi Arabia

**Keywords:** end‐stage renal disease, hydrothorax, peritoneal dialysis, pleural effusion, pleuroperitoneal fistula

## Abstract

Hydrothorax is a serious complication of peritoneal dialysis, and it may be resolved by deferring dialysis or decreasing dialysate volumes. Repeat thoracentesis is not well tolerated in children. Therefore, if conservative measures fail, thoracotomy or thoracoscopy with endoscopic repair of associated diaphragmatic eventration should be considered before reinstating peritoneal dialysis.

## 1 INTRODUCTION

Secondary diaphragmatic eventration with massive hydrothorax is an unusual and rare complication of peritoneal dialysis (PD), with few reported cases. However, isolated hydrothorax is relatively common and can be seen in 1.6%‐10% of PD patients.^1^ Usually, these patients present with acute breathlessness and a decrease in the ultrafiltration rate the in case of massive hydrothorax. However, mild effusion may remain asymptomatic, or the patient may complain of a dry cough.^2^ Massive hemothorax usually results in temporal or permanent discontinuation of PD. The problem may be resolved by deferring PD or decreasing dialysate volumes; severe cases may require invasive surgical intervention. We report the case of a patient with left hemidiaphragm eventration and massive hydrothorax who underwent diagnostic thoracoscopy with successful laparoscopic repair.

## 2 CASE EXAMINATION

An 11‐year‐old boy was diagnosed with a case of Galloway‐Mowat syndrome at the age of six when he presented with developmental delay, intellectual disability, clumsy gait, microcephaly, hypotonia, complex partial seizure, and nephrotic range proteinuria. Due to a history of the same complaints in the patient's younger siblings, an extensive genetic workup was performed, and the results revealed a homozygous mutation of NUP107: NM_020401.2:c.303G> A: P. (Met101Ile). A renal biopsy showed features of collapsing focal segmented glomerulosclerosis (FSGS).

The patient progressed to end‐stage renal disease (ESRD) within a few years. Peritoneal dialysis was initiated successfully. He was discharged on PD with the following prescription: Dianeal^®^ 1.36%, filling volume of 1000 mL/m^2^, 10 cycles for 10 hours with last fill of Extraneal 500 mL/m^2^.

The patient presented to the emergency room with an acute episode of coughing and shortness of breath approximately 6 weeks after starting PD. Peritoneal dialysis was running with many low drain volume alarms, and there was no history of fever, cloudy PD drain, or abdominal pain. On physical examination, the patient was tachypneic, and a chest examination showed signs of effusion on the left side of the chest.

## 3 DIFFERENTIAL DIAGNOSIS, INVESTIGATIONS AND TREATMENT

**Figure 1:**
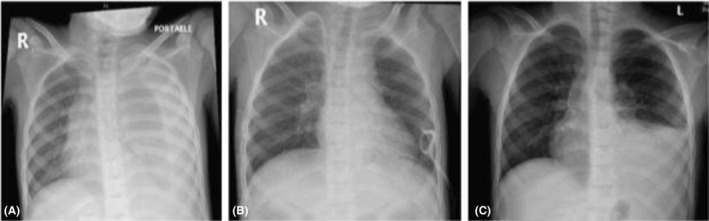
The patient's chest X‐ray showed massive left‐sided hydrothorax (A). A repeat chest X‐ray examination performed after reinstitution of peritoneal dialysis showed the hydrothorax was almost cleared (B). A chest X‐ray performed after one month revealed a recurrence of the hydrothorax on the left side (C)

A chest X‐ray was requested, and it showed massive left‐sided effusion (Figure 1A). The patient underwent urgent pigtail drainage during which 1200 mL of fluid was drained, and a sample was sent for laboratory analysis. The results were as follows: glucose concentration 15.5 mmol/L (279 mg/dL), protein 0.52 g/L, and white blood cell 20/cu mm, with polymorphonuclear rate of 10%. Fluid culture and polymerase chain reaction for the most common infective organism were negative. Serum albumin was normal and a cardiology work‐up was unremarkable. Peritoneal dialysis was deferred, and intermittent hemodialysis (IHD) was offered.

A pleuroperitoneal fistula was suspected after consulting a radiologist, and due to the limited availability of a confirmatory diagnostic test, the decision was taken to resume PD after clearing the effusion, and the pigtail was kept in place.

## 4 OUTCOME AND FOLLOW‐UP

Serial chest X‐ray examinations were performed up to two weeks after resuming PD, and the effusion was almost cleared (Figure 1B). A chest X‐ray performed after one month revealed a recurrence of the hydrothorax on the left side, estimated to be moderate, and the patient was asymptomatic (Figure 1C). Peritoneal dialysis was discontinued, and the patient was switched again to chronic IHD.

Due to vascular access insufficiency and the incompatible social contingency associated with IHD, the patient's case was discussed with a pediatric surgeon regarding the repair of the pleuroperitoneal fistula and reinstitution of PD. The patient underwent diagnostic/therapeutic thoracoscopy after 1200 mL/m^2 ^of dialysis solution (Dianeal 1.36% mixed with methylene blue dye) was instilled into the peritoneal cavity one hour before the surgery. A full exploration of the diaphragm revealed no leakage of the dialysate mixed dye from the peritoneal to the pleural cavity. Instead, there was huge central bulging of a muscular left hemidiaphragm above the nipple line, which was repaired with interrupted suturing of the eventuated diaphragm. We were able to resume PD one week post‐operative without the recurrence of the effusion, as evidenced on serial chest X‐rays, up to six weeks post‐surgery.

## 5 DISCUSSION

This case report addresses hydrothorax in patients on continuous cycling peritoneal dialysis (CCPD). Hydrothorax is a relatively rare complication observed in about 2% of PD patients,^3^, ^4^, ^5^ and it is either related to a pleuroperitoneal fistula with leakage of dialysis fluid from the peritoneal cavity into the pleural cavity or can be secondary to diaphragmatic eventration. Several mechanisms, such as congenital diaphragmatic defects or channel, pleuroperitoneal pressure gradients, and lymph drainage disorders may explain the leakage,^5^ but severe muscular hypotonia and an underlying neurological problem are important contributing factors for associated diaphragmatic eventration in our patient. Patients with massive hydrothorax commonly present with shortness of breath, cough, and chest pain. Some patients with hydrothorax might be completely asymptomatic, and the condition may be detected fortuitously if it is mild. Hydrothorax is almost always seen on the right side, as reported in published cases; however, our patient developed left‐sided hemothorax, which is not completely explained by the associated diaphragmatic eventration.^6^ This complication usually results in either temporary or permanent discontinuation of PD.

Peritoneal dialysis‐related hydrothorax should be suspected and managed early in any PD patient presenting with signs of effusion; however, most diagnostic imaging modalities have limited utility in these cases. Computed tomographic peritoneography, which is a commonly used diagnostic tool, is noninvasive and useful in cases of large pleuroperitoneal communication, but it is not sensitive in detecting smaller defects. Radionuclide scans (such as Tc‐99m diethylene‐triamine‐pentaacetate) are increasingly being used to confirm peritoneal‐pleural leakage. These scans have sensitivity rates ranging between 40% and 50%, but they still have a limited utility in locating the site of the fistula.^7^ The diagnosis of PD‐related hydrothorax in our patient was clear‐cut based on the recurring nature of the effusion and the results of biochemical tests. Consequently, we proceeded with thoracoscopy. The decision to go for a more invasive and interventional procedure was influenced by the fact that PD was the only suitable long‐term dialysis modality for our patient. In addition, diagnostic thoracoscopy has a high diagnostic accuracy and therapeutic potential.

The treatment of PD‐related hydrothorax due to peritoneal‐pleural fistula can be either conservative or interventional depending on the severity of the effusion and the associated diaphragmatic abnormalities. Success rates of up to 60% have been reported, with patients being able to resume maintenance PD.^7^, ^8^ In cases where PD cannot be reinstituted, the patient should be permanently switched to chronic hemodialysis. Different management strategies have been suggested to treat peritoneal‐pleural leakages and resume PD. Conservative treatment, including deferment of PD for six weeks to three months or decreasing dialysate volumes, was found to be successful in up to 53% of PD patients.^9^ Recent reports have demonstrated the effectiveness of conservative therapy in patients who developed PD‐related hydrothorax.^10^, ^11^ In both reports, the PD prescription was altered to normal volume daytime ambulatory PD transiently before continuous ambulatory PD was resumed a few months later. Long‐term resolution of the hydrothorax was achieved with no sign of recurrence several years later, suggesting that normal volume daytime ambulatory was a potentially appealing and cheap method for the resolution of pleura‐peritoneal fistula.

Failure of conservative treatment or massive hydrothorax is an indication for more invasive treatment. Repeat thoracentesis has been effective in adults, but it is not well tolerated in children. Therefore, if conservative measures fail, thoracotomy or thoracoscopy with endoscopic repair of associated diaphragmatic eventration can be curative in up to 60% of patients, in whom PD can be reinstituted. Adkins et al described three patients on continuous ambulatory peritoneal dialysis with massive hydrothorax. Two patients had right hydrothorax and one presented with a left hydrothorax. Diaphragmatic eventrations were detected in all three cases, and the patients underwent surgical repair through thoracotomy. All three patients resumed PD successfully.^4^

## CONFLICT OF INTEREST

None declared.

## AUTHOR CONTRIBUTION

This Manuscript is single authored.
